# Predictive value of serum inflammatory markers in retinopathy of prematurity

**DOI:** 10.1038/s41433-024-03260-5

**Published:** 2024-07-25

**Authors:** Eşay Kıran Yenice, Caner Kara, Tijen Karsli Türkoglu, Dilek Ulubaş Işık, İstemi Han Çelik

**Affiliations:** 1 Department of Ophthalmology, University of Health Sciences, Etlik Zübeyde Hanım Maternity and Women’s Health Teaching and Research Hospital, Ankara, Turkey; 2 Department of Ophthalmology, Etlik City Hospital, Ankara, Turkey; 3 Department of Neonatology, University of Health Sciences, Etlik Zübeyde Hanım Maternity and Women’s Health Teaching and Research Hospital, Ankara, Turkey

**Keywords:** Eye diseases, Retinal diseases

## Abstract

**Purpose:**

To evaluate the relationship between the development of retinopathy of prematurity (ROP) and neutrophil-to-lymphocyte ratio (NLR), lymphocyte-to-monocyte ratio (LMR), and platelet-to-lymphocyte ratio (PLR).

**Material and methods:**

The medical records of 153 preterm infants born before the 34th week of gestation, were retrospectively reviewed. Complete blood cell (CBC) and C-reactive protein (CRP) results measured within the first 24 h of life were recorded. NLR, LMR and PLR were calculated by dividing neutrophil count by lymphocyte count, lymphocyte count by monocyte count, and platelet count by lymphocyte count, respectively. Analysis of possible risk factors related with ROP development was evaluated using logistic regression analysis. Results were compared between infants with and without ROP.

**Results:**

A total of 153 infants, of which 64 (41.9%) with ROP and 89 (58.1%) without ROP, were included in the study. While lymphocyte count and LMR were found to be significantly lower in infants with ROP (*p* = 0.015 and *p* = 0.044), neutrophil count and NLR were found to be significantly higher (*p* = 0.021 and *p* = 0.046, respectively). No significant difference were observed in platelet and monocyte count and PLR (*p* = 0.808, *p* = 0.170 and *p* = 0.075, respectively). Multivariate logistic regression analysis revealed that gestational age, birth weight and NLR were major risk factors for the development of ROP (OR:0.59; *p* = 0.01, OR:1.00; *p* = 0.02 and OR: 2.56; *p* = 0.02, respectively).

**Conclusion:**

This study supports that, in addition to prematurity, NLR on the first postnatal day has a significant predictive value in ROP.

## Introduction

Retinopathy of Prematurity (ROP) is a proliferative vascular disorder of the developing retina, characterized by retinal ischemia and neovascularization, which limits the development of retinal vessels in premature infants. As the survival chances of premature infants increase thanks to advances in neonatal care, the development of ROP, which can cause vision problems and blindness, also increases [1, 2]. Although many etiologic factors are associated with the development of ROP, the mechanisms in the pathogenesis are not fully known. Considering the pathophysiology of ROP, it is reported that angiogenic, oxidative and neuroprotective factors and cytokines affected by hyperoxia and hypoxia phases contribute to the development of ROP [3–7]. Moreover, many studies report that neonatal inflammation is associated with the development of ROP by playing a role in both normal and pathological angiogenesis in the retina [8, 9]. High white blood cell (WBC) and C-reactive protein (CRP) levels have been implicated to be a strong prognostic factor in predicting the development of ROP and response to treatment [10]. Furthermore, WBC ratios have been proposed as potential inflammatory indicators in a variety of diseases, including cancer, coronary artery disease, kidney disease, gynaecological diseases, and eye diseases [3, 6, 11–16].

In previous studies, these parameters were evaluated in the field of ROP, but the examination criteria, analysed WBC ratios and evaluation times of postnatal blood samples and results differ from each other. In the present study, we aimed to evaluate the relationship between development of retinopathy of prematurity (ROP) and neutrophil-to-lymphocyte ratio (NLR), lymphocyte-to-monocyte ratio (LMR), and platelet-to-lymphocyte ratio (PLR) in the first 24 h of life.

## Material and methods

The Ethical Review Committee approved this study (2023/01), which performed in line with the standards of the Declaration of Helsinki for research involving human subjects. The medical records of 153 preterm infants born before the 34th week of gestation, who were screened for ROP in our neonatal intensive care unit (NICU) and outpatient clinic between January 2021 and December 2022, were retrospectively reviewed. Infants with blood culture-proven sepsis, necrotizing enterocolitis (NEC) and haematological disease, and the infants who received blood product transfusion and postnatal steroid treatment were excluded from the study. The infants’ gender, gestational age (GA), birth weight (BW), postmenstrual age (PMA) at examination, ROP Stages and Zones and treatment options were recorded. Infants were divided into two groups as infants with and without ROP. Initial ROP screening was carried out at the 4 to 6 weeks after birth for all infants. The examination was performed one hour after pupil dilatation with 0.5% tropicamide (Tropamid, Bilim İlaç, Turkey) and 2.5% phenylephrine (Mydfrin, Alcon, USA). Following topical anaesthesia with 0.5% proparacaine hydrochloride (Alcaine, Alcon, USA), fundus examination was performed using a binocular indirect ophthalmoscope with 20 D and/or 28 D lenses, a paediatric eye speculum and a scleral depressor. ROP findings of the infants were categorized according to the International Classification of ROP, third edition (ICROP-3) criteria, based on zone, stage, extent of the disease, and the presence or absence of plus disease [17]. Follow-up examinations of the infants were adjusted according to the presence and severity of ROP. Treatment of the infants was implemented according to the Early Treatment for Retinopathy of Prematurity (ETROP) [18] and Bevacizumab Eliminates the Angiogenic Thread of ROP (BEAT-ROP) [19] studies.

Risk factors that may affect the development of ROP such as mode of delivery, multiple pregnancy, bronchopulmonary dysplasia (BPD; oxygen requirement >36 weeks PMA), respiratory distress syndrome (RDS), hypoxic ischemic encephalopathy (HIE), intraventricular haemorrhage (IVH), patent ductus arteriosus (PDA, requiring treatment), small for GA (SGA; < 10th percentiles), duration of invasive mechanical ventilation (days), total days on oxygen, maternal preeclampsia, gestational diabetes mellitus and prelabour rupture of the membranes (PROM) were recorded for each infant. Due to the possibility of infection that may affect the clinical course, and the need for postnatal steroid and blood transfusion, complete blood cell (CBC) and CRP results measured within the first 24 h of life were evaluated. NLR, LMR and PLR were calculated by dividing neutrophil count by lymphocyte count, lymphocyte count by monocyte count, and platelet count by lymphocyte count, respectively.

### Statistical analysis

Statistical analysis was performed with the Statistical Package for the Social Sciences (SPSS Inc., Chicago, Illionis, USA) version 25.0. Categorical data were presented as number (n) and percentage (%), and descriptive data were presented as mean ± standard deviation (SD). Normality of the distribution of continuous variables was evaluated using the Kolmogorov–Smirnov test. In pairwise comparisons of variables, independent samples t test was used for normally distributed data, and Mann–Whitney *U* test was used for non-normally distributed data. Categorical data were analysed using the Chi-square test. Logistic regression analysis was used to estimate the association between risk factors and ROP development. *P*-values of 0.05 or less were considered to indicate statistical significance. Odds ratios (OR) and 95% confidence intervals (CIs) were calculated for each risk factor. Additionally, the receiver operating characteristic (ROC) curve produced by plotting between sensitivity and 1-spesificity was performed to analyse the NLR for ROP prediction and summarized by the area under the curve (AUC).

## Results

One hundred fifty-three preterm infants born before the 34th week of gestation were included in the study. Of the 153 infants, 82 (53.6%) were female and 71 (46.4%) were male. Overall, 132 of the infants had a history of caesarean section (C/S) and it was significantly lower in the infants with ROP (*p* = 0.002). Among all infants, 64 (41.8%) infants had ROP; mean GA was 28 ± 1 week (24–33 weeks) and mean BW was 1161 ± 209 g (700 to 1870 g). GA and BW were significantly lower in infants with ROP (*p* = 0.000 for both GA and BW). Of these, 37 (57.8%) developed stage I ROP, 27 (42.2%) stage II ROP, and no treatment was required in these infants. PMA at examination in infants with ROP was 32.26 ± 1.44 weeks (range: 28–37 weeks) and in infants without ROP was 34.23 ± 1.85 weeks (range: 30–39 weeks), respectively (*p* = 0.000). Additionally, as expected, the prevalence of RDS, IVH, PROM, PDA and total days on oxygen were more common in infants with ROP (*p* = 0.001, *p* = 0.013, *p* = 0.025, *p* = 0.05 and *p* = 0.000, respectively).

CBC parameters such as haemoglobin (Hb) (16.90 ± 2.62) and haematocrit (Hct) (50.59 ± 7.56) values were lower and WBC (10.82 ± 6.49) and CRP (1.08 ± 4.42) was higher in infants with ROP. While lymphocyte count (47.72 ± 17.22) and LMR (6.69 ± 4.33) were found to be significantly lower in infants with ROP (*p* = 0.015 and *p* = 0.044), neutrophil count (40.08 ± 16.22) and NLR (1.17 ± 1.06) were found to be significantly higher (*p* = 0.021 and *p* = 0.046). No significant difference was observed in platelet (254.59 ± 70.03; 257.53 ± 76.11) and monocyte (9.34 ± 4.55; 8.40 ± 4.80) count and PLR (6.23 ± 3.27; 5.00 ± 1.77) between infants with and without ROP (*p* = 0.808, *p* = 0.170 and *p* = 0.075). Demographic data, risk factors and the first 24 h blood count values of infants with and without ROP are presented in Table 1.

**Table 1:** Demographic data, risk factors and blood count values within the first 24 h of life of infants with and without retinopathy of prematurity (ROP).

		ROP (+) (*n* = 64)	ROP (−) (*n* = 89)	*P* value
Gestational age (weeks)	Mean ± SD	28 ± 1	30 ± 2	**<0.001**^**b**^
Birth weight (g)	Mean ± SD	1161 ± 209	1455 ± 345	**<0.001**^**b**^
Gender (Female)	*n*, %	38 (44%)	44 (47.2%)	0.224^a^
Multiple pregnancy	*n*, %	17 (26.5%)	33 (37%)	0.171^a^
Postmenstrual age at examination (weeks)	Mean ± SD	32.26 ± 1.44	34.23 ± 1.85	**<0.001**^**b**^
Mode of delivery: C/S	*n*, %	56 (87.5%)	76 (85.4%)	**0.002**^**a**^
White blood cell count (×10^3 µL)	Mean ± SD	10.82 ± 6.49	9.14 ± 3.62	0.299^b^
C-reactive protein (mg/L)	Mean ± SD	1.08 ± 4.42	0.33 ± 0.81	0.203^b^
Haemoglobin (g/dL)	Mean ± SD	16.90 ± 2.62	17.20 ± 2.09	0.777^b^
Haematocrit	%	50.59 ± 7.56	51.78 ± 6.11	0.284^b^
Neutrophil count (×10^3 µL)	Mean ± SD	40.08 ± 16.22	34.74 ± 12.04	**0.021**^**b**^
Lymphocyte count (×10^3 µL)	Mean ± SD	47.72 ± 17.22	53.69 ± 12.76	**0.015**^**b**^
Monocyte count (×10^3 µL)	Mean ± SD	9.34 ± 4.55	8.40 ± 4.80	0.170^b^
Platelet count (×10^3 µL)	Mean ± SD	254.59 ± 70.03	257.53 ± 76.11	0.808^b^
NLR	Mean ± SD	1.17 ± 1.06	0.74 ± 0.41	**0.046**^**b**^
LMR	Mean ± SD	6.69 ± 4.33	8.35 ± 5.54	**0.044**^**b**^
PLR	Mean ± SD	6.23 ± 3.27	5.00 ± 1.77	0.075^b^
BPD (oxygen requirement >36 weeks PMA)	*n*, %	2 (3.1%)	5 (5.6%)	0.467^a^
Respiratory distress syndrome	*n*, %	59 (92.2%)	62 (69.6%)	**0.001**^**a**^
Hypoxic ischemic encephalopathy	*n*, %	1 (1.6%)	–	0.237^a^
Intraventricular haemorrhage	*n*, %	5 (7.8%)	–	**0.013**^**a**^
Patent ductus arteriosus (requiring treatment)	*n*, %	21 (32.8%)	17 (19.1%)	**0.05**^**a**^
SGA < 10^th^ Percentile	*n*, %	1 (1.6%)	3 (3.4%)	0.489^a^
Prelabour rupture of the membranes	*n*, %	15 (23.4%)	9 (10.1%)	**0.025**^**a**^
Maternal preeclampsia	*n*, %	8 (12.5%)	7 (7.9%)	0.342^a^
Gestational diabetes mellitus	*n*, %	5 (7.8%)	12 (13.5%)	0.271^a^
Duration of invasive mechanical ventilation (days)	Mean ± SD	0.89 ± 2.06	1.30 ± 2.89	0.375^b^
Total days on oxygen	Mean ± SD	24.13 ± 18.39	14.44 ± 17.41	**<0.001**^**b**^

Bold, statistically significant values are highlighted.

*C/S* cesarean section, *NLR* neutrophil-to-lymphocyte ratio, *LMR* lymphocyte-to-monocyte ratio, *PLR* platelet-to-lymphocyte ratio, *BPD* bronkopulmoner dysplasia, *SGA* small for gestational age, *SD* standard deviation;

^a^Chi-square test.

^b^Mann-Whitney *U* test.

**Fig. 1:**
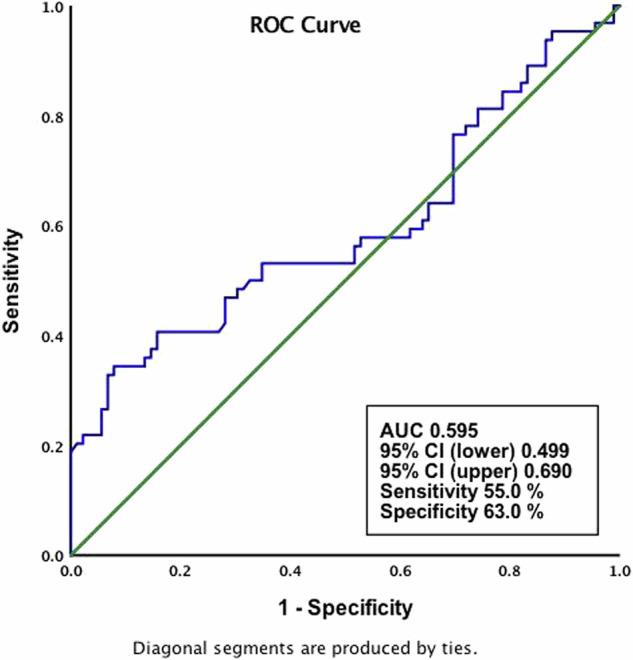
Receiver-operating characteristics (ROC) curve analyses of neutrophil-to-lymphocyte ratio for predicting ROP. AUC Area under the curve.

Multivariate logistic regression analysis revealed that GA, BW and NLR were the major risk factors for the development ROP (OR:0.59; 95% CI: 0.38–0.90; *p* = 0.01, OR:1.00; 95% CI: 0.99–1.00; *p* = 0.02 and OR:2.56; 95% CI: 1.65–399.91; *p* = 0.02, respectively) (Table 2). Each variable has been shown to be significant for the development of ROP. The ROC curve of NLR for ROP prediction, representing the relationship between sensitivity and 1-specificity, is shown in Fig. 1. The AUC for NLR was 0.595 (95% CI:0.499–0.690), and a NLR count of 8.24 or greater predicted ROP with a sensitivity of 55% and specificity of 63%.

**Table 2:** Multivariable logistic regression analysis of risk factors related with ROP development.

	Multivariable analysis	
	OR (95% CI)	*P* value
Gestational age	0.59 (0.38–0.90)	0.01
Birth weight	1.00 (0.99–1.00)	0.02
NLR	2.56 (1.65–399.91)	0.02

## Discussion

The current study were observed significantly higher neutrophil count and NLR in infants with ROP. Additionally, NLR was found to be a major factor in predicting the development of ROP as well as prematurity.

It is known that inflammatory mediators and cells have a significant role in the pathogenesis of ROP. Systemic inflammatory stress can affect retinal functions by causing abnormal retinal vascularization and an increase in vascular anastomoses and predispose preterm infants to ROP [7]. Many studies have shown that NLR, which measures the effects of neutrophilia, a marker of inflammation, and lymphopenia, a marker of physiological stress, is a valuable prognostic marker in patients with systemic inflammation [13, 20, 21]. Kurtul et al. showed that NLR and lymphocyte count were associated with ROP in infants born before the 32nd week of gestation, and that lymphocyte count was inversely associated with ROP, although NLR did not appear to be an independent predictor [3]. Guthrie et al. also suggested that NLR may be a greater indicator of inflammation than the total leukocyte count [20]. In the present study, it was observed that neutrophil count and NLR were significantly higher in infants with ROP, and the predictive effect of NLR on ROP continued in multivariate analysis as well as in univariate analysis. Additionally, ROC curve was generated to determine the cut-off value of NLR in predicting ROP. Our cut-off value was 8.24. This result shows that premature infants with NLR of 8.24 or greater have a higher risk of ROP. However, due to the small number of infants in the groups and the close distribution of NLR values in the groups, it was observed that the sensitivity (55%) and specificity (63%) values of NLR in predicting ROP were low and although significant, it affected the regression analysis results. Significance needs to be confirmed with a larger sample. Additionally, similar to this study, lymphocyte count was found to be significantly lower in infants with ROP.

Also, like NLR, LMR, which reflects the balance between lymphocytes and monocytes migrating from the bone marrow to the peripheral blood during inflammation, has been shown to be an indicator of the inflammatory response in many diseases [22, 23]. While Çelik et al. observed a non-significant inverse relationship between LMR and ROP, Hu et al. and Akdoğan et al. stated that the relationship between the development of ROP and LMR was significant [6, 16, 22]. In present study, as well as lymphocyte count, LMR were significantly lower in infants with ROP. In addition, a non-significant but higher monocyte count was detected in infants with ROP. On the other hand, there are studies indicating that, in addition to NLR and LMR, thrombocytopenia is also associated with ROP [24–26]. PLR has also been suggested to be a prognostic indicator of systemic inflammation in some tumours [27, 28]. In present study, although the platelet count was lower and PLR was higher in infants with ROP, no significant relationship was found in terms of ROP development.

There are also studies in the literature evaluating the relationship between WBC and ROP with different results [6, 16, 26]. Although the WBC count was found to be higher in infants with ROP in our study, it was not significant. Exclusion of conditions that may cause infection and affect the clinical course, such as culture-positive sepsis and NEC, but the presence of factors that may affect intrauterine inflammatory reactions such as PROM, gestational diabetes, and preeclampsia may have affected the results.

Limitations of our study include its retrospective design, small sample size, absence of advanced-stage ROP infants, and lack of details regarding maternal anaemia and postnatal weight gain. Due to small number of infants, the overall severity of ROP was low and also we did not have any infants with advanced stage ROP who required treatment. Therefore, the current study does not provide insight into infants with advanced ROP. Future studies with more patients are needed to evaluate the prognostic value of serum inflammatory markers in the development of ROP.

In conclusion, our findings reflect that, in addition to prematurity, NLR on the first postnatal day has a predictive value in the development of ROP and that early evaluation of NLR levels may be useful for predicting ROP in premature infants.

## Summary

### What was known before

- In previous studies, serum inflammatory markers were evaluated in the field of ROP, but the examination criteria, analysed WBC ratios and evaluation times of postnatal blood samples and results differ from each other.

### What this study adds

- Our findings reflect that, in addition to prematurity, neutrophil-to-lymphocyte ratio (NLR) on the first postnatal day has a predictive value in the development of ROP and that early evaluation of NLR levels may be useful for predicting ROP in premature infants.

## Data Availability

The dataset used and analysed during the current study are available from the corresponding author on reasonable request.
