# Correction: Predictive value of serum inflammatory markers in retinopathy of prematurity

**DOI:** 10.1038/s41433-024-03293-w

**Published:** 2024-08-09

**Authors:** Eşay Kıran Yenice, Caner Kara, Tijen Karsli Türkoglu, Dilek Ulubaş Işık, İstemi Han Çelik

**Affiliations:** 1 Department of Ophthalmology, University of Health Sciences, Etlik Zübeyde Hanım Maternity and Women’s Health Teaching and Research Hospital, Ankara, Turkey; 2 Department of Ophthalmology, Etlik City Hospital, Ankara, Turkey; 3 Department of Neonatology, University of Health Sciences, Etlik Zübeyde Hanım Maternity and Women’s Health Teaching and Research Hospital, Ankara, Turkey

**Keywords:** Eye diseases, Retinal diseases

Correction to: *Eye* 10.1038/s41433-024-03260-5, published online 25 July 2024

In the original version, the author Dilek Ulubaş Işık was mistakenly assigned with affiliations 1 and 3 whereas only affiliation 3 is correct. The correct affiliation is “Department of Neonatology, University of Health Sciences, Etlik Zübeyde Hanım Maternity and Women’s Health Teaching and Research Hospital, Ankara, Turkey”. The article has been corrected.

